# A very long and winding road: developing novel therapeutics for metastatic tumors

**DOI:** 10.18632/oncoscience.595

**Published:** 2024-02-09

**Authors:** Paul Dent

**Keywords:** chemotherapy, MET, immunotherapy, evolutionary resistance mechanisms, lung cancer

*“Regrettably, these proposals have not been implemented in clinical practice. The standard practice involves continuing monotherapy until tumor progression occurs, at which point all cells express R1 resistance. And, in most cases, the game is lost”* [1].

*“Unfortunately, I was not treated with preemptive combinations. This approach seems too weird to standard oncologists: targeting the invisible, or preventing resistance before this resistance is detectable by any means, the sooner, the better”* [1].

Tumors that have metastasized to distant locations, such as the brain, are most often impossible to treat and cure, although immunotherapeutic approaches have had recent successes in some tumor types such as NSCLC and cutaneous melanoma. There is, however, also considerable evidence that immune therapy may cause hyper-progression in some NSCLC patients, potentially including Dr. Blagosklonny, whose tumor comprises METex14 and amplification of MDM2, as well as in melanoma and NHSCC patients. There are several issues that presently preclude more effective control of solid tumors both *in situ* and as metastatic disease. The first is that the mutations which drive a cancer phenotype are generally the combination of subtle alterations in cell biology, any one of which, if targeted, *if it can be targeted*, will only have modest effects on tumor growth and survival. Conceptually, this calls for an immediate use of two- and three-drug combinations blocking key signaling pathways to achieve effective tumor control regardless of whether resistance mechanisms evolve. Second, a corollary of altered cell biology, and highlighted in the article, is that fewer tumors have a single recognizable driving oncogene to which the tumor cell is specifically addicted for growth and survival, e.g., mutant RAS proteins, mutant EGF receptors and other mutant receptors of MET, RET, and HER2. And even under these circumstances based on a large body of evidence from the past 20 years is that such tumors also require treatment with two- and three-drug combinations that simultaneously interdict the primary driving oncogene, block signaling from the primary evolving resistance mechanism and even block signaling from a secondary survival pathway.

Given that the MET inhibitor capmatinib caused a remarkable response in Dr. Blagosklonny, a pertinent question remains as to why he was not treated with “preemptive drug combinations” to kill the tumor and to simultaneously prevent the development of capmatinib resistance (see Figures 6 and 7 in [1]). And therein lies one of the thorniest problems in developing novel drug combination concepts from the bench to the bedside to widespread clinical use. Taking a concept itself, first to the bench and then to the bedside may take two years, with the subsequent modestly expensive phase I safety trial on 12–25 patients an additional two years. If the phase I trial shows a “signal” in a specific tumor cell type, and a large amount of money is available, a phase II trial can take place which may take three years and enroll 50–100 patients. Even at this point, the novel combination needs to be compared to the standard-of-care therapy for the disease in a phase III trial, i.e., the combination will not receive approval if it cannot show a better progression free survival and overall survival than standard-of-care. Thus, to answer the initial rhetorical question: in an ideal world to increase therapeutic options for oncologists and their patients, it could be argued that in the future a novel drug combination which is shown to be safe and has broad anti-cancer effects in a diverse range of tumor types should be “fast-tracked” through the standard approvals process, facilitating the rapid delivery of novel drug combinations to cancer patients.

For some tumor types where rapid morbidity and mortality are evident regardless of any standard-of-care therapy, such as in pancreatic cancer, glioblastoma or uveal melanoma, the process of moving from the bench to the bedside and trials can be accelerated as any improvement in the quality of life or extended survival can be more rapidly obtained and quantified. In contrast, in diseases such as breast cancers and non-small cell lung cancers, where there are already multiple efficacious modalities to prolong survival with a good quality of life, it will be more difficult to bring novel drug combination concepts to the clinic.

When thinking about the development of novel therapies, particularly when trying to subvert resistance mechanisms, it is beneficial to keep in mind several rough guidelines. (1) Are any of the drugs in your novel combination FDA approved? (2) If not, how far along the development pipeline are your drugs? (and is the drug company financially stable or running out of cash). (3) Are the drugs approved for adults or for children? (4) Are the drugs FDA approved for the specific malignancy you are studying? (USA insurance = billing!) (5) If not, are the drugs at least compendium listed for the malignancy you are studying? (6) Do the drugs in your combination have overlapping or separate normal tissue toxicities? For example, tyrosine kinase inhibitors often have GI toxicities which could be dose-limiting for a two-drug combination. (7) Talking with the MSL persons from the drug companies to define an initial level of interest? In the case of performing studies with drugs from two drug companies the issue arises whether the companies “like” each other and how willing they are to collaborate: for example, will the owners of afatinib (Boehringer-Ingleheim) collaborate with the owners of capmatinib (Novartis/Incyte) to collaborate on a phase I trial?

Finally, it is worth noting that many drug companies are developing agents that specifically inhibit the activated (mutant) form of growth factor receptors, and perhaps the best known is osimertinib for the treatment of mutated EGFR NSCLC. Unlike patients who had previously been treated with gefitinib, where mouth sores, GI upset and general malaise were observed, osimertinib treated patients maintain a good quality of life. There is, however, a ‘catch’ with such specifically targeted drugs, namely that tumors can also become resistant to drugs not by expressing a novel mutated receptor but by simply over-expressing wild type receptors, and with an autocrine ligand to keep a low level of activity. In this instance, would a drug such as osimertinib achieve significant long-term tumor control? No doubt several groups are already addressing this question.

## Abbreviations

EGFR: epidermal growth factor receptor
NSCLC: non-small cell lung cancer
MDM2: Mouse double minute 2 homolog
NHSCC: neck and head squamous cell carcinoma
